# Multi-Transcript Level Profiling Revealed Distinct mRNA, miRNA, and tRNA-Derived Fragment Bio-Signatures for Coping Behavior Linked Haplotypes in HPA Axis and Limbic System

**DOI:** 10.3389/fgene.2021.635794

**Published:** 2021-08-19

**Authors:** Kevin Gley, Frieder Hadlich, Nares Trakooljul, Fiete Haack, Eduard Murani, Ulrike Gimsa, Klaus Wimmers, Siriluck Ponsuksili

**Affiliations:** ^1^Leibniz Institute for Farm Animal Biology (FBN), Institute of Genome Biology, Dummerstorf, Germany; ^2^Leibniz Institute for Farm Animal Biology (FBN), Institute of Behavioral Physiology, Dummerstorf, Germany

**Keywords:** coping behavior, HPA axis, limbic system, sncRNA, mixomics, transcriptomics, tRF, tiRNA

## Abstract

The molecular basis of porcine coping behavior (CB) relies on a sophisticated interplay of genetic and epigenetic features. Deep sequencing technologies allowed the identification of a plethora of new regulatory small non-coding RNA (sncRNA). We characterized mRNA and sncRNA profiles of central parts of the physiological stress response system including amygdala, hippocampus, hypothalamus and adrenal gland using systems biology for integration. Therefore, ten each of high- (HR) and low- (LR) reactive pigs (*n* = 20) carrying a CB associated haplotype in a prominent QTL-region on SSC12 were selected for mRNA and sncRNA expression profiling. The molecular markers related to the LR group included *ATP1B2*, *MPDU1*, miR-19b-5p, let-7g-5p, and 5′-tiRNA^*Leu*^ in the adrenal gland, miR-194a-5p, miR-125a-5p, miR-7-1-5p, and miR-107-5p in the hippocampus and *CBL* and *PVRL1* in the hypothalamus. Interestingly, amygdalae of the LR group showed 5′-tiRNA and 5′-tRF (5′-tRF^*Lys*^, 5′-tiRNA^*Lys*^, 5′-tiRNA^*Cys*^, and 5′-tiRNA^*Gln*^) enrichment. Contrarily, molecular markers associated with the HR group encompassed miR-26b-5p, tRNA^*Arg*^, tRNA^*GlyiF*^ in the adrenal gland, *IGF1* and *APOD* in the amygdala and *PBX1*, *TOB1*, and *C18orf1* in the hippocampus and miR-24 in the hypothalamus. In addition, hypothalami of the HR group were characterized by 3′-tiRNA enrichment (3′-tiRNAGln, 3′-tiRNA^*Asn*^, 3′-tiRNA^*Val*^, 3′-tRF^*Pro*^, 3′-tiRNA^*Cys*^, and 3′-tiRNA^*Ala*^) and 3′-tRFs enrichment (3′-tRF^*Asn*^, 3′-tRF^*Glu*^, and 3′-tRF^*Val*^). These evidence suggest that tRNA-derived fragments and their cleavage activity are a specific marker for coping behavior. Data integration revealed new bio-signatures of important molecular interactions on a multi-transcript level in HPA axis and limbic system of pigs carrying a CB-associated haplotype.

## Introduction

Situations that induce a physiological stress reaction (e.g., psychosocial stress in intensive pig production settings) require an adaptive strategy in order to minimize the damaging outcomes of chronic stress, including decreased fertility and growth rates as well as compromised immune competence (Moberg and Mench, 2000; Proudfoot and Habing, 2015; Gimsa et al., 2018). Shedding light upon the molecular roots of this process called coping is a challenging scientific endeavor, since functional traits like behavioral characteristics have a highly complex underlying genetic and epigenetic basis. Evolution has produced diverging adaptive response patterns to external stressors (coping styles) that can broadly be distinguished into a pro-active or active and a re-active or passive coping style. Differences between the pro-active and the re-active coping style are increased levels of aggression and territorial control as well as a more pronounced sympathetic nervous system activity in the pro-active type. The activity of the hypothalamic-pituitary-adrenal (HPA)-axis is moderate in the pro-active and high in the re-active coping style (Koolhaas et al., 1999, 2010).

In our previous studies, we analyzed differential mRNA expression between two haplotypes in a coping behavior associated QTL region in the four tissue types adrenal gland, hypothalamus, amygdala and hippocampus (Gley et al., 2019a,b). In a further study, we used a deep sequencing approach to explore small non-coding RNA (sncRNA) expression in central parts of the physiological stress and anxiety response system in the same animals (Haack et al., 2019). The non-coding fraction of the transcriptome consists of several types of RNA molecules which share the characteristic that they are not translated into a protein. Functionally important types of non-coding RNA (ncRNA) include transfer RNAs (tRNAs), ribosomal RNAs (rRNAs), long ncRNAs as well as the group of small non-coding RNAs (sncRNAs). MicroRNAs (miRNAs) are short (20–24 nts) evolutionary highly conserved sncRNAs that affect stability as well as translation of their mRNA targets via base-pairing (Bartel, 2004). Binding to the 3′-UTR of the mRNA decreases its stability and terminates protein translation. Alternatively, the miRNA can prevent translation by inhibition of 5′-cap reading (Liu et al., 2014). It is estimated that up to 60% of mammalian genes are targeted by miRNAs, hence they are considered to be key players in post-transcriptional gene expression regulation (Friedman et al., 2009). Recent studies found evidence that a multitude of miRNAs are highly expressed throughout the central nervous system where they are considered to be crucial regulators of processes like cell proliferation, differentiation and apoptosis as well as neuronal protection, development and synaptic plasticity (Krichevsky, 2007; Bak et al., 2008). tRNAs act as mRNA-decoding adapter molecules in gene translation. Additionally to this traditional function, emerging research indicated that tRNAs form a major source of regulatory sncRNA displaying a variety of distinct functions (Anderson and Ivanov, 2014). tRNA-derived sncRNA can be categorized into tRNA-derived stress-induced RNA (tiRNA), also known as tRNA halves, and tRNA-derived fragments (tRFs). Importantly, both tRNA-derived sncRNAs are not generated by random degradation but rather are the result of precisely coordinated biogenetic processes (Anderson and Ivanov, 2014). tiRNAs are specific cleavage products (29–50 nt long 5′-tRNA and 3′-tRNA halves) which are formed by angiogenin action under various stress conditions like starvation, hypoxia and oxidative stress (Yamasaki et al., 2009). With a length of 16–28 nts, tRFs are shorter tRNA-derived fragments. Depending on their sites of origin, they are classified into tRF-5, tRF-3, tRF-1, and i-tRF fragments (Anderson and Ivanov, 2014). Beyond their canonical role, both tRF as well as tiRNAs can perform a variety of biological functions including cell signaling and survival processes, apoptosis, amino acid and porphyrine metabolism, and stress response programs (Phizicky and Hopper, 2010; Raina and Ibba, 2014). These diverse roles are performed by acting as sncRNAs in different ways: They can exert RNA interference in a fashion similar to miRNAs, directly inhibit protein synthesis by eIF4G translation initiation factor displacement, regulate target mRNA stability by protein factor binding and modulate apoptosis in concert with cytochrome C (Gebetsberger et al., 2012; Sobala and Hutvagner, 2013; Saikia et al., 2014). Stress-related functions of tiRNAs and tRFs are the assembly of stress granules and p53 linked oxidative-stress sensitization and apoptosis (Hanada et al., 2013). Further noticeable sncRNA species include yRNA, piRNA as well as snoRNA.

Previous studies focused primarily on the identification of small subsets of molecules for the explanation of phenotypes and, moreover, used univariate approaches, where each biological feature is considered independently. Based on our previous work, which showed that coping behavior was associated with a prominent QTL region on SSC12 (Ponsuksili et al., 2015) and individuals of each group are characterized by specific gene expression patterns in all four tissues (Gley et al., 2019a,b), we hypothesize that integration of mRNA and sncRNA data measured on the same animals reveal distinct group-specific biomarker panels. We combined large-scale biological data sets including microarray mRNA as well as deep sequencing sncRNA expression data for the discovery of novel molecular insights underlying different coping behavior phenotypes, adding to the knowledge of the complex interplay between different transcriptomic layers.

## Materials and Methods

### Ethics Approval, Health, and Safety

Animal care and tissue collection procedures followed the guidelines of the German Law of Animal Protection and the experimental protocol was approved by the Animal Care Committee of the Leibniz Institute for Farm Animal Biology as well as by the State Mecklenburg-Western Pomerania (Landesamt für Landwirtschaft, Lebensmittelsicherheit und Fischerei; LALLF M-V/TSD/7221.3-2.1-020/09). The experimental protocol was carried out in accordance with the approved guidelines for safeguarding good scientific practice at the institutions in the Leibniz Association and the measures were taken to minimize pain and discomfort and accord with the guidelines laid down by the European Communities Council Directive of 24 November 1986 (86/609/EEC). All mandatory laboratory health and safety procedures have been complied within the course of conducting any experimental work reported in our study.

### Animals Selection, Coping Behavior Assessment, and Sample Collection

The animals used in this study were selected from a bigger pool consisting of 294 German Landrace (DL) piglets, which were used in our previous GWAS (Ponsuksili et al., 2015). Inclusion criteria for the present study are phenotypic extremity for early life coping behavior trait as well as the haplotypes at a coping behavior-associated QTL (Gley et al., 2019a,b). Using the same animals, early life coping style and haplotype-based classification allowed the selection of 20 pigs—10 high reactive (HR) animals and 10 low reactive (LR) animals. Early life coping behavior phenotype was assessed using backtests according to Zebunke et al. (2017). Early life backtest behavior reflects the coping strategy and is a heritable and repeatable indicator also at later age. Our previous study including a much larger data set of 3,555 animals, of which the animals used here represent a subset, could demonstrate that the correlations among the backtest traits frequency, duration and latency of struggles were moderate to high at a single time point (*r*_*s*_ = | 0.63–0.78|) and moderate between each trait at different time points (*r*_*s*_ = | 0.19–0.44|). Genetic correlations were high for all traits and time points (*r*_*g*_ > 0.89). Backtests were conducted on days 5, 12, 19, and 26 *post natum*. In brief, piglets were swiftly put onto their backs in a cellulose-padded V-shaped device. After reaching immobility, a 60 s test period started. Recorded backtest parameters included Latency (interval between initial immobility and first struggling attempt), Duration (total time spent struggling) and Frequency (count of escape attempts). In a subsequent step, the total scores of latency (tL), duration (tD), and frequency (tF) were determined by summing up the individual parameters on days 5, 12, 19, and 26 *post natum*. For a detailed description of haplotype estimation and trend regression analysis (Gley et al., 2019a). As described in our previous study, average 157 days post natum, experimental animals were weighed, stunned by electronarcosis and slaughtered by exsanguination (Gley et al., 2019a). Immediately after exsanguination, adrenal glands were collected and sampling of intra-cranial structures was carried out by swiftly removing the pig’s brain. The amygdala (including sub-nuclei), the hypothalamus and the hippocampus were anatomically localized and excised using a stereotaxic atlas of the porcine brain as reference guide. Tissue samples were flash-frozen in liquid nitrogen and stored at –80°C for transcriptome profiling.

### Microarray Data

We used microarray data from our previous studies (Gley et al., 2019a,b). In brief, whole porcine transcriptomes of amygdala, adrenal gland, hippocampus and hypothalamus were analyzed using Affymetrix snowball arrays (Affymetrix, Santa Clara, CA, United States). The amplified sense strand cDNA for microarray hybridization was generated using the Ambion WT Expression Kit (Ambion, Austin, TX, United States). The cDNA fragmentation and biotin-labeling steps were carried out using Affymetrix GeneChip WT Terminal Labeling Kit (Affymetrix, Santa Clara, CA, United States) and individual samples were hybridized on the gene chips. Following staining, washing and scanning of the arrays, data was processed using Affymetrix GCOS 1.1.1 software. The raw data was deposited in the NCBI Gene Expression Omnibus^1^ (GEO: GSE125079, GSM3562317-GSM3562336; GSE125080, GSM3562337-GSM3562356; GSE109155, GSM293170-2933189, and GSM2933190-2933209). Raw data was normalized by the robust multichip average algorithm (RMA) and further pre-processed using the detection above background (DABG) algorithm in Affymetrix Expression Console 1.3.1.187 (Affymetrix, Santa Clara, CA, United States). Merely probes that were present in at least 80% of the total number of samples were kept. These normalized and filtered data was transformed into a mixOmics compatible numeric data matrix.

### Illumina Hiseq Next-Generation Deep Sequencing Data

In our previous study (Haack et al., 2019), raw sncRNA deep sequencing datasets were converted to FASTQ format and submitted to ArrayExpress. It can be accessed at http://www.ebi.ac.uk/arrayexpress (accession number: E-MTAB-7499). We used this data in our present study and applied the following further processing steps: Adapter trimming and filtering of contaminated sequences as well as low-quality reads using Flexbar v3.2 and FastQC v0.11.5 was followed by removal of low abundance reads (<10 reads/sequence). The produced reads are referred to as clean reads. The number of reads (in millions) and mapping statistics from all samples have been reported previously (Haack et al., 2019). Briefly, in amygdala, hippocampus, hypothalamus, and adrenal gland, there are 101, 99, 139, and 59 M unique reads, respectively, with an average of 95% mapped reads in the porcine genome (Ssc11.1). Where necessary, unique sequence reads were generated by collapsing identical sequence reads within one set of clean reads. CCA sequences that were attached to tRNA 3′ tails during post-transcriptional processing were removed before mapping of the sequences. In a next step, we used the bowtie aligner to map the reads to *sus scrofa* genome RefSeq assembly 11.1 (GCF_000003025.6). Two mismatches were allowed and the best mapped reads according to the “best strata” mode were retained. *Sus scrofa* annotation release 106 was used for annotation and complemented by miRDeep2 for high confidence annotation of existing miRNA as well as discovery of putative novel miRNA (Friedlander et al., 2012). In order to further illuminate the identity of non-miRNA sncRNAs, we consulted the high confidence annotation set provided by Anthon et al. (2014). Additionally, we carried out a homology-based database matching with the pan-specific RNAcentral database^2^ (Petrov et al., 2017). RNAcentral assesses mapping quality regardless of taxonomic similarity. Since there is a higher level of ncRNA conservation among taxonomically closely related species, we evaluated the confidence of RNAcentral results against this background.

### Integration of mRNA, miRNA, and tRNA Data

We used the R package MixOmics v6.8.5 to integrate Omics data. This tool provides an array of multivariate methods for exploration, integration as well as visualization of large-scale biological datasets from different sources (Rohart et al., 2017). Our input dataset comprised microarray mRNA expression data as well as NGS miRNA and tRNA data measured on the same 20 samples. mRNA data were normalized, log2 transformed and filtered. The tRNA as well as miRNA read count matrices were transformed and normalized using the DESeq2 method “variance-stabilizing transformation” (VST) (Love et al., 2014). All input files were converted to mixOmics compatible numeric data matrices and the Data Integration Analysis for Biomarker Discovery (DIABLO) using Latent cOmponents implementation was employed (Singh et al., 2019). DIABLO is able to integrate complex data sets of heterogenerous origin generated by different platforms and measured on different scales. Since high throughput biological data integration produces multiple highly correlated variables, we used the sparse partial least squares regression discriminant analysis (sPLS-DA) for variable selection (Lê Cao et al., 2011). sPLS-DA represents a natural extension to the classic sPLS proposed by Lê Cao et al. (2008). We applied the mixOmics *block.splsda()* function for the identification of highly correlated variables (X) which simultaneously explain the categorical variable (Y) used to supervise the analysis. To assess the number of parameters, the global performance, the balanced error rate (BER), and to select the optimal metric distance and define the number of components kept for our block.splsda analysis, we computed the evaluation criteria using the perf() function from DIABLO. As input arguments we used our block.splsda object (without variable selection), Mfold validation (*n* = 10), repeated the cross-validation (50 repetitions), and calculated the area under the curve (AUC). We fine-tuned our model using tune.block.splsda() function, and determined the optimal number of variables kept for our final block.splsda analysis and the downstream analysis. We ran tune.block.splsda() using the results from our perf() run indicating the use of mahalanobis distance, two components and two cross-validation steps (nrepeat = 2). We then applied the mixomics block.splsda() function using the our data as input, three components, and the features to select from each component. We created performance plots in order to observe the overall balanced error rate (BER) and determine the optimal number of components. The centroids.dist graph seems to produce the best accuracy. The output variable $choice.ncomp integrates the centroids.dist distance as well as the BER and indicates the optimal number of components for the final DIABLO model. On average across all four tissues, the greatest decrease of the BER occurred from first to second component, hence we decided to select 2 as optimal number of components. We visualized these components of the *block.splsda* results using the *plot.loadings()* function. This function creates a horizontal bar plot visualization of loading vectors. Each variable’s contribution to each component is depicted in a bar plot where length of the bars correspond to the loading weight (representing importance) of the feature. For discriminant analysis, highest or lowest median values of the variables with color code corresponding to the outcome of interest are visualized, where color corresponds to the group in which the feature is most abundant. Additionally, a circos plot was created from the *block.splsda* results, depicting the highest and lowest Pearson’s correlations between most discriminant mRNAs, miRNAs and tRNAs. Relevant associations between the X and Y variables were displayed using the *network()* function, by using a pairwise association score. To achieve improved clarity of the depicted network, a threshold score (cut-off) of 0.80 was set in order to exclusively represent variables X and Y with an association score greater than that threshold. In the resulting network, each X- and Y-variable corresponds to a node and the edges portray the association between them. Finally, all identified molecules linked to coping behavior haplotypes in each tissue were mapped to the pig genome (Sscrofa 11.1) using the R package *circlize* (Gu et al., 2014).

### Prediction of miRNA Targets and Correlation Between miRNA and mRNA Profiles

Based on Ensembl annotation version 101, 17064 3′-UTR sequences, 16857 5′-UTR sequences and 20320 coding sequences were extracted from the *Sus scrofa* genome (Sscrofa11.1). Extracted sequences were split into 2 kb fragments with a 50 base overlap. The outputs were considered as potential base pairing targets to the given miRNA using RNAhybrid version 2.1.2 with a binding energy cut-off of –25 k, a helix constraint ranging from 2 to 7, and one hit per target setting (Krüger and Rehmsmeier, 2006). Each potentially hybridizing miRNA-mRNA pairing is summarized by its minimum free energy and its *p*-value. The mRNA expression data of the same samples was used for a pairwise correlation analysis. Pearson correlations between miRNA and mRNA profiles of the same samples in each tissue were calculated and considered significant at FDR < 5%. Only negatively correlating miRNA/mRNA pairs were included in further analyses and subjected to IPA software (Ingenuity Systems, Redwood City, CA) for functional analysis. This software categorizes genes based on annotated gene functions and statistically tests for over-representation of functional terms within the gene list. Benjamini-Hochberg *p*-value adjustment was applied using cut-off level FDR < 0.05.

### Validation of miRNA NGS Data With Quantitative Real-Time PCR

The cDNA synthesis of selected miRNAs was performed according to Mentzel et al. (2014). Briefly, 1 unit of poly(A) polymerase 1 μM was used to attach poly(A) tails to 250 ng of small RNA. The product was reverse transcribed using RT-primers (CAGGTCCAGTTTTTTTTTTTTTTTVN where V equals A, C, and G and N equals A, C, G, and T), 0.1 mM of NTPs and 100 units of MuLV reverse transcriptase (Invitrogen, Carlsbad, CA, United States). The synthetic cDNA spike-in miR-39-1 was added to the RT mix to monitor RT efficiency variance. The RT mix was incubated at 42°C for 1 h. Subsequently, enzymatic activity was terminated by a 95°C step. In total, expression of 12 miRNAs from 80 individual samples was investigated using the Fluidigm BioMark HD qPCR System (Fluidigm Corporation, San Francisco, CA). Specific target amplification (STA) was conducted following manufacturer’s recommendations. In a next step, pre-amplification sample mixtures with a total volume of 5 μL, containing 1.25 μL of cDNA, 1 μL PreAmp Master Mix (Fluidigm PN 1005581), and 0.5 μL Pooled Delta Gene Assay Mix (500 nM) were composed. After incubation of pre-amplification mixtures, exonuclease I treatment as well as 10 × dilution of STA with DNA suspension buffer (TEKnova, PN T0221) followed. Quantitative real-time measurements were carried out using 96.96 (96 samples × 96 assays) dynamic arrays (Fluidigm Corporation, CA, United States) as instructed by the manufacturer. Resulting data were analyzed with proprietary real-time PCR analysis software in the BioMark HD instrument. The miRNAs 5S and cel-miR-39-3p were used as references and calculations were based on the 2−ΔCt method. Primer sequences were shown in Supplementary Table 1. Pearson correlation coefficient (r) calculations between the NGS miRNA data and qPCR data were performed using the *rcorr ()* function in R.

## Results

Tissue samples of porcine adrenal gland, amygdala, hippocampus and hypothalamus using microarray-based mRNA expression profiling as well as sncRNA deep sequencing from our previous studies were used for downstream analyses (Gley et al., 2019a,b; Haack et al., 2019). Discovered sncRNA structures mainly consisted of miRNA, Rrna, and tRNA, with additional low abundance RNA species including yRNA, piRNA as well as snoRNA. All data sets were measured on the same samples and were normalized and filtered as well as integrated by using mixOmics in order to create coping haplotype related biomarker panels.

### Integration and Identification of a Biomarker-Related Coping Style Haplotype

After filtering of NGS data, in total 363 miRNAs and 173 tRNAs including their cleavage products were retained and used for subsequent analyses in all tissues. Numbers of post-filter analysis ready mRNAs were 11,837 for the adrenal gland, 9,312 for the amygdala, 9,769 for the hippocampus and 10,795 in case of the hypothalamus. We applied the sPLS-DA function of the mixOmics R package for the identification of relationships between highly discriminant mRNAs, miRNAs, and tRNAs separately for all four tissues. The optimal bio-signature of different transcript types in adrenal, amygdala, hippocampus, and hypothalamus was identified via 2 components. In addition, these identified markers were also correlated with each other, as indicated by a positive or negative relationship.

**FIGURE 1:**
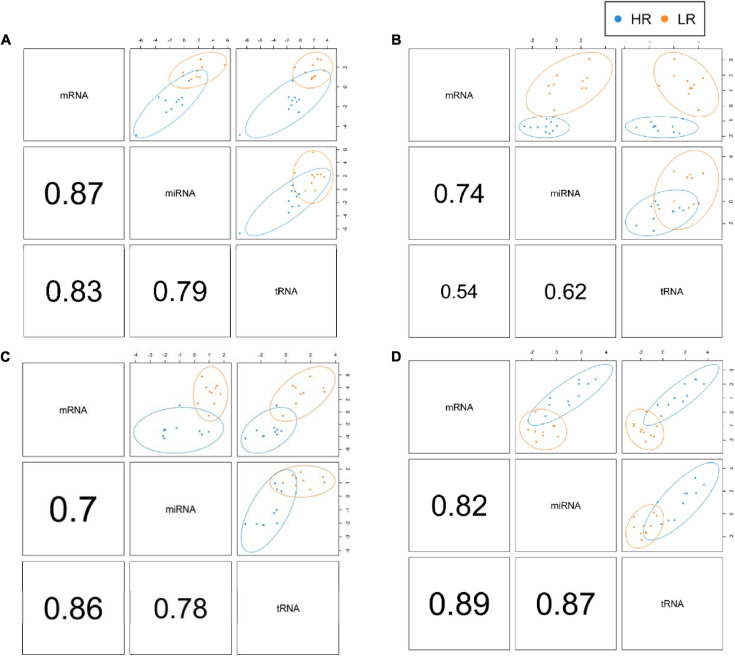
DIABLO plots of the adrenal gland **(A)**, amygdala **(B)**, hippocampus **(C)** and hypothalamus **(D)** showing individual correlation strengths between the different RNA pairs. Red color indicates low-reactive (LR) group and blue depicts high-reactive (HR) group association.

**FIGURE 2:**
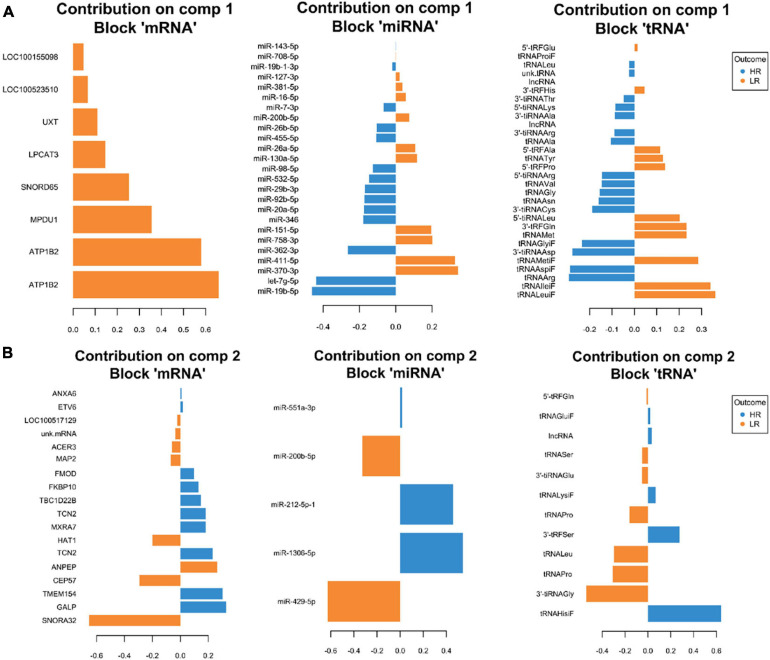
Representation of the optimal biomarker panel of mRNA, miRNA, and tRNA correlated to coping behavior haplotype groups over two component sets in the adrenal gland. DIABLO minimal set of feature selection across data types could discriminate between high-reactive (HR) and low-reactive (LR) groups in component 1 **(A)** and component 2 **(B)**. Blue-colored bars are indicators for the HR group, yellow-colored bars indicate the LR group (DIABLO: Data Integration Analysis for Biomarker discovery using Latent cOmponents).

**FIGURE 3:**
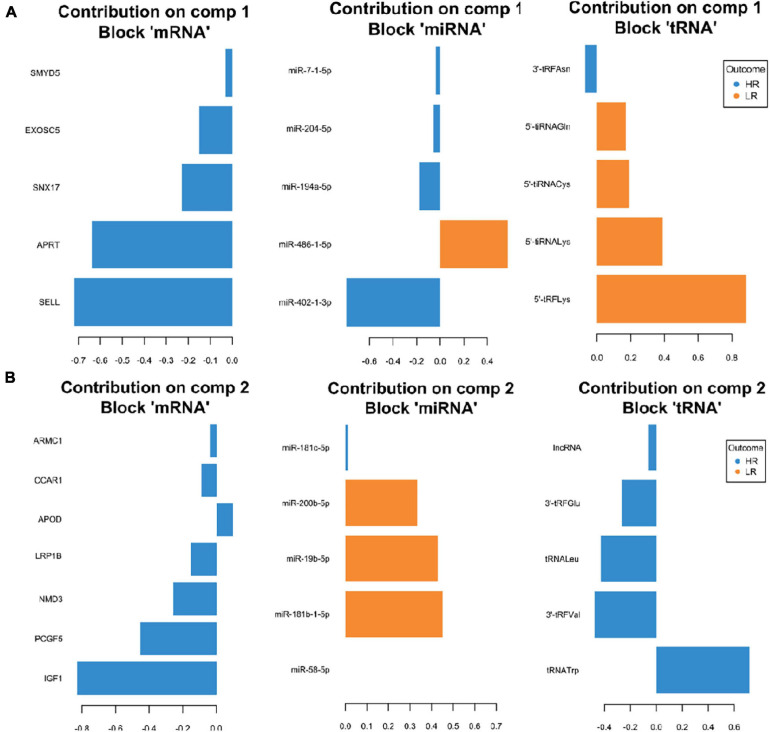
Representation of the optimal biomarker panel of mRNA, miRNA and tRNA correlated to coping behavior haplotype groups over two component sets in the amygdala. DIABLO minimal set of feature selection across data types could discriminate between high-reactive (HR) and low-reactive (LR) groups in component 1 **(A)** and component 2 **(B)**. Blue-colored bars are indicators for the HR group, yellow-colored bars indicate the LR group (DIABLO: Data Integration Analysis for Biomarker discovery using Latent cOmponents).

**FIGURE 4:**
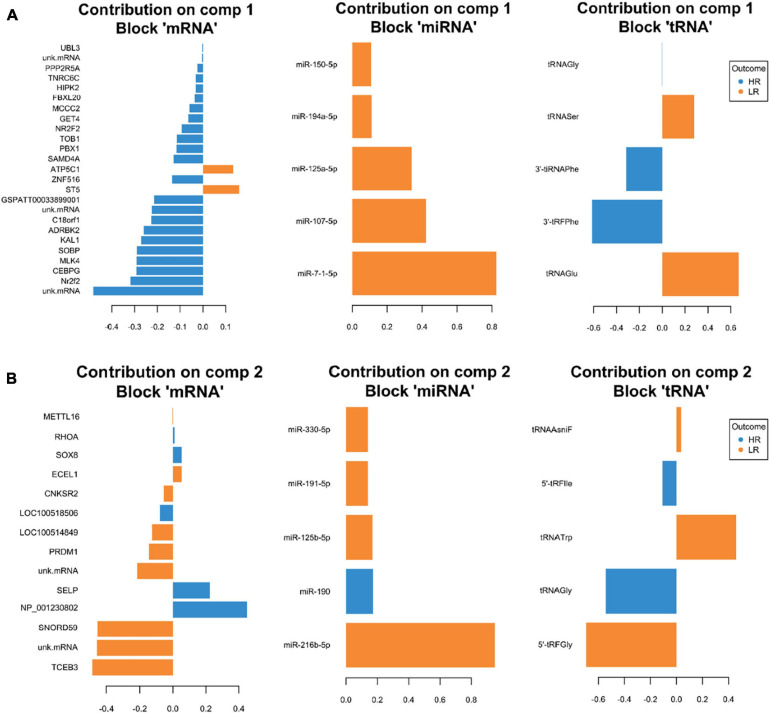
Representation of the optimal biomarker panel of mRNA, miRNA, and tRNA correlated to coping behavior haplotype groups over two component sets in the hippocampus. DIABLO minimal set of feature selection across data types could discriminate between high-reactive (HR) and low-reactive (LR) groups in component 1 **(A)** and component 2 **(B)**. Blue-colored bars are indicators for the HR group, yellow-colored bars indicate the LR group (DIABLO: Data Integration Analysis for Biomarker discovery using Latent cOmponents).

**FIGURE 5:**
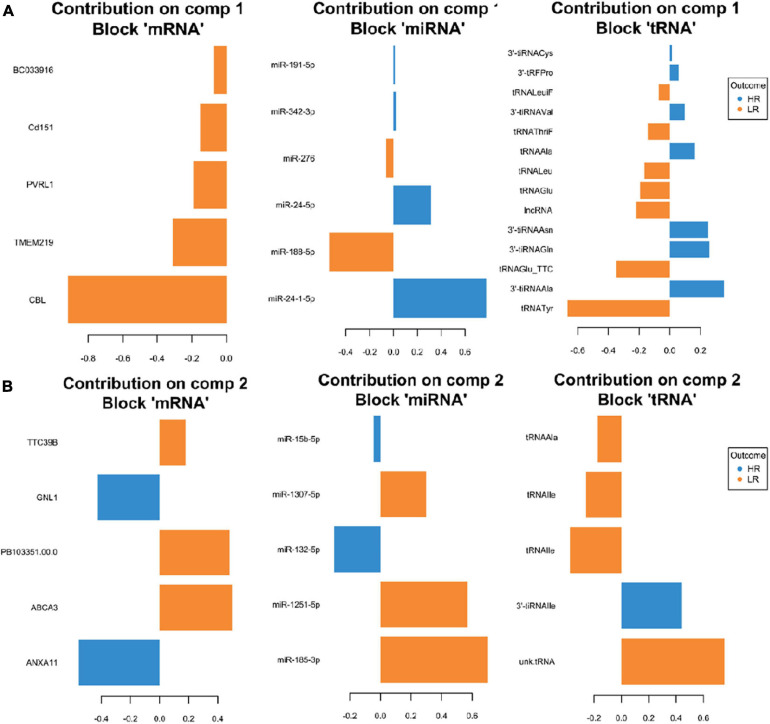
Representation of the optimal biomarker panel of mRNA, miRNA, and tRNA correlated to coping behavior haplotype groups over two component sets in the hypothalamus. DIABLO minimal set of feature selection across data types could discriminate between high-reactive (HR) and low-reactive (LR) groups in component 1 **(A)** and component 2 **(B)**. Blue-colored bars are indicators for the HR group, yellow-colored bars indicate the LR group (DIABLO: Data Integration Analysis for Biomarker discovery using Latent cOmponents).

**FIGURE 6:**
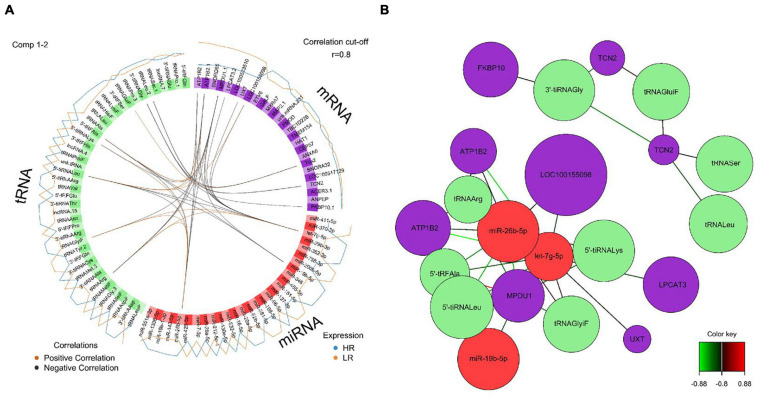
**(A)** Circos plot of mRNA, miRNA, and tRNA linked to coping behavior haplotype groups in the adrenal gland. Positive correlations are indicated by brown-colored connectors, while black connectors indicate negative correlation between mRNA, miRNA and tRNA. Blue and orange colored lines in the outer circle highlight expression levels in either high (HR) or low (LR) reactive coping group. **(B)** Network visualization of mRNA, miRNA, and tRNA panels indicating correlated variables in the adrenal gland (*r* > | 0.80|). Purple colored nodes represent mRNA, red nodes indicate miRNA and green nodes highlight tRNA biomarkers. Correlation between nodes is indicated by edge color as shown in the color key. Negative correlation is depicted by green edges, while red edges represent positive correlation.

**FIGURE 7:**
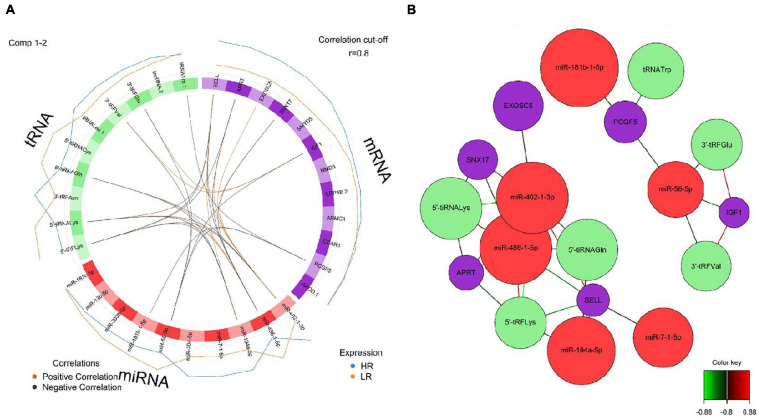
**(A)** Circos plot of mRNA, miRNA, and tRNA linked to coping behavior haplotype groups in the amygdala. Positive correlations are indicated by brown-colored connectors, while black connectors indicate negative correlation between mRNA, miRNA, and tRNA. Blue and orange colored lines in the outer circle highlight expression levels in either high (HR) or low (LR) reactive coping group. **(B)** Network visualization of mRNA, miRNA and tRNA panels indicating correlated variables in the amygdala (*r* > | 0.80|). Purple colored nodes represent mRNA, red nodes indicate miRNA and green nodes highlight tRNA biomarkers. Correlation between nodes is indicated by edge color as shown in the color key. Negative correlation is depicted by green edges, while red edges represent positive correlation.

**FIGURE 8:**
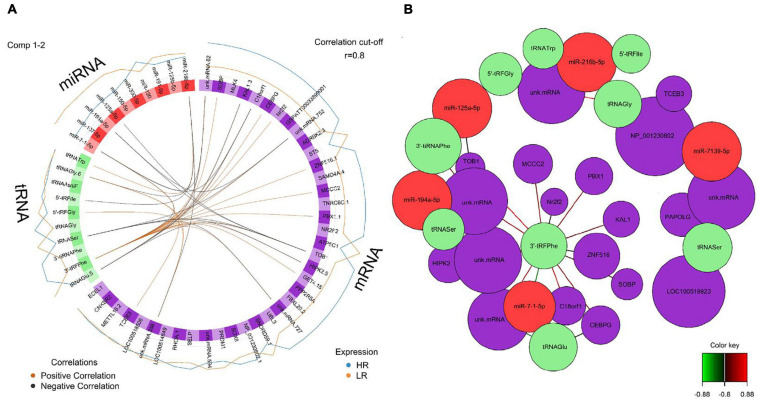
**(A)** Circos plot of mRNA, miRNA, and tRNA linked to coping behavior haplotype groups in the hippocampus. Positive correlations are indicated by brown-colored connectors, while black connectors indicate negative correlation between mRNA, miRNA, and tRNA. Blue and orange colored lines in the outer circle highlight expression levels in either high (HR) or low (LR) reactive coping group. **(B)** Network visualization of mRNA, miRNA and tRNA panels indicating correlated variables in the hippocampus (*r* > | 0.80|). Purple colored nodes represent mRNA, red nodes indicate miRNA and green nodes highlight tRNA biomarkers. Correlation between nodes is indicated by edge color as shown in the color key. Negative correlation is depicted by green edges, while red edges represent positive correlation.

**FIGURE 9:**
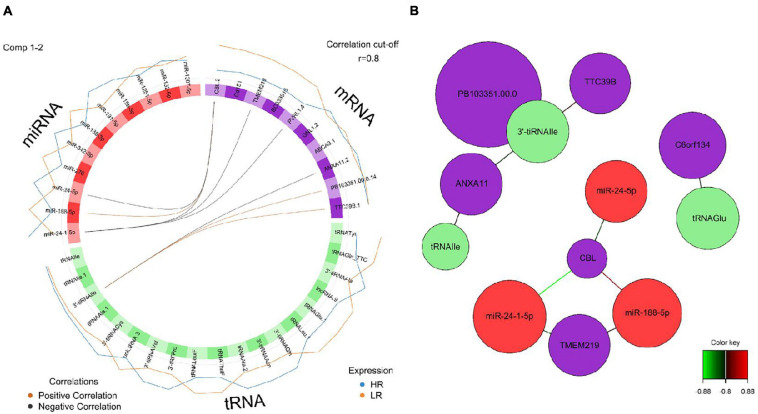
**(A)** Circos plot of mRNA, miRNA, and tRNA linked to coping behavior haplotype groups in the hypothalamus. Positive correlations are indicated by brown-colored connectors, while black connectors indicate negative correlation between mRNA, miRNA and tRNA. Blue and orange colored lines in the outer circle highlight expression levels in either high (HR) or low (LR) reactive coping group. **(B)** Network visualization of mRNA, miRNA, and tRNA panels indicating correlated variables in the hypothalamus (*r* > | 0.80|). Purple colored nodes represent mRNA, red nodes indicate miRNA and green nodes highlight tRNA biomarkers. Correlation between nodes is indicated by edge color as shown in the color key. Negative correlation is depicted by green edges, while red edges represent positive correlation.

**FIGURE 10:**
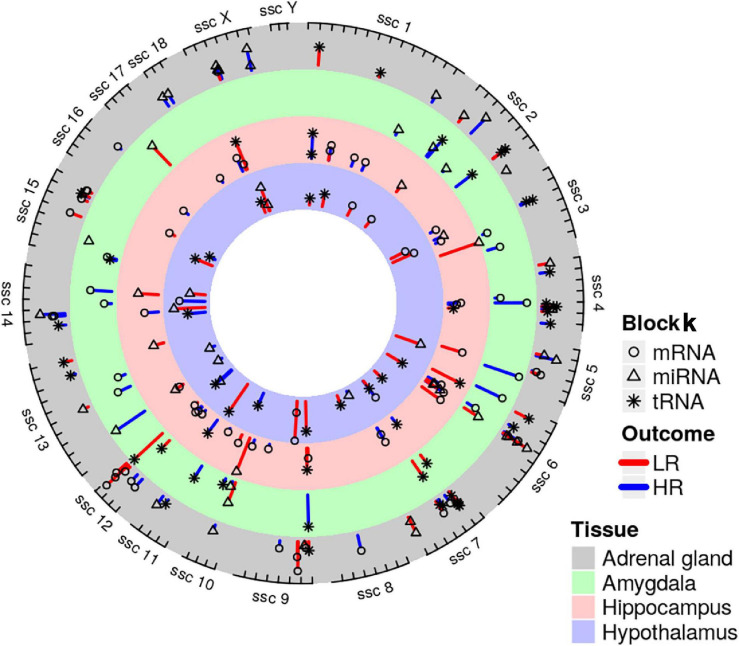
Genomic positions of mRNA (depicted as circularly shaped), miRNA (triangularly shaped) and tRNA (star-shaped) coping behavior related molecules derived by DIABLO analysis of adrenal gland, amygdala, hippocampus and hypothalamus. Molecules were mapped to the Sus scrofa reference genome. Coping behavior haplotype group association is indicated by line color and its length, which illustrates the variation (red highlights the low-reactive (LR) and blue indicates the high-reactive (HR) group).

We created DIABLO plots to demonstrate the correlation strengths between the different RNA types [Figure 1; Adrenal gland (a), amygdala (b), hippocampus (c), hypothalamus (d)]. Strong correlations ranging from *r* = 0.79 to *r* = 0.87 could be observed between mRNA and miRNA, mRNA and tRNA and miRNA and tRNA in the adrenal gland. The corresponding RNA pairs in the other tissues were moderately to highly correlated: 0.54 ≤ *r* ≤ 0.74 in the amygdala, 0.70 ≤ *r* ≤ 0.86 in the hippocampus and 0.82 ≤ *r* ≤ 0.89 in the hypothalamus. Next, we used the *plotLoadings()* function to present the contributions of the mRNAs, miRNAs and tRNAs that were associated with each group on the first and second component for each tissue. The contributions on components 1 and 2 of block “mRNA,” “miRNA,” and “tRNA” were shown in Figures 2A,B for the adrenal gland, Figures 3A,B for the amygdala, Figures 4A,B for the hippocampus and Figures 5A,B for the hypothalamus. The *CircosPlot()* function using the correlation cutoff | *r*| > 0.8 was applied to illustrate the correlation between the various omics blocks for each tissue (Adrenal gland: Figure 6A, amygdala: Figure 7A, hippocampus: Figure 8A, hypothalamus: Figure 9A). The relevance networks created by the *network()* function depict the correlation levels between the top molecules. At cutoff level 0.80, the adrenal network (Figure 6B) consisted of seven mRNAs (*TCN2*, *FKBP10*, *ATP1B2*, *MPDU1*, *LPCAT3*, *UXT*, and *LOC100155098*), three miRNAs (miR-19b-5p, miR-26b-5p, and let-7g-5) and nine tRNAs (tRNA^*Arg*^, 5′-tiRNA^*Lys*^, tRNA^*GlyiF*^, 5′-tiRNA^*Leu*^, 5′-tRF^*Ala*^, tRNA^*Leu*^, tRNA^*Ser*^, tRNA^*GluiF*^, 3′-tiRNA^*Gly*^). In the amygdala network (Figure 7B), *SELL* was identified as biomarker linked to coping behavior. While correlated miRNAs including miR-194a-5p, miR-7-1-5p, miR-402-1-3p showed positive correlation, the tRNA molecules 5′-tRF^*Lys*^ and 5′-tiRNA^*Gln*^ were negatively correlated to *SELL*. Other strong coping behavior associated biomarkers in this subnetwork comprised the mRNAs *SNX17*, *APRT*, *EXOSC5* the miRNAs miR-486-1-5p and 5′-tiRNA^*Lys*^. *IGF1* mRNA was connected to miR-58-5p, 3′-tRF^*Val*^ and 3′-tRF^*Glu*^. The mRNA of *PCGF5* was linked to miR-181b-1-5p and miR-59-5p as well as tRNA^*Trp*^. In total 19 mRNAs at cutoff level 0.8 were included in the hippocampus network (Figure 8B). Important examples were *TOB1*, *HIPK2*, *SOBP*, *MCCC2*, *KAL1*, *PAPOLG*, *PBX1*, *NR2F2*, *TCEB3*, and *ZNF516*. miR-125a-5p, miR-194-5p, miR-7139-5p as well as miR-216b-5p represented the miRNA share of the hippocampus network while the eight tRNAs included 3′-tiRNA^*Phe*^, tRNA^*Ser*^, 3′-tRF^*Phe*^, 5′-tRF^*Gly*^, 5′-tRF^*IIe*^, tRNA^*Gly*^, tRNA^*Trp*^, and tRNA^*Glu*^. Prominent mRNA biomarkers in the hypothalamus network (Figure 9B) included *TTC39B*, *C6orf134*, *ANXA11*, *TMEM219*, and *CBL*. Additionally, three miRNAs, miR-188-5p, miR-24-5p and miR-24-1-5p as well as the three tRNAs 3′-tiRNA^*IIe*^, tRNA^*IIe*^ and tRNA^*Glu*^ were found in the panel. The mixOmics framework DIABLO constructs components as linear combinations of mRNA, miRNA and tRNA which are maximally correlated across all input data types with a specific outcome variable—in our study the high and low -reactive coping behavior groups. Simultaneous minimal marker selection associated with the outcome groups is performed. The optimal different transcript types bio-signature in adrenal gland, amygdala, hippocampus and hypothalamus was identified in this study consisting of in total 228 molecules including 86 mRNA, 61 miRNA, and 81 tRNA and their cleavage products across two component sets (Supplementary Table 2). The whole set of molecules was mapped to the pig genome as shown in Figure 10 and Supplementary Table 1.

### miRNA Bio-Signature and Target Prediction

**FIGURE 11:**
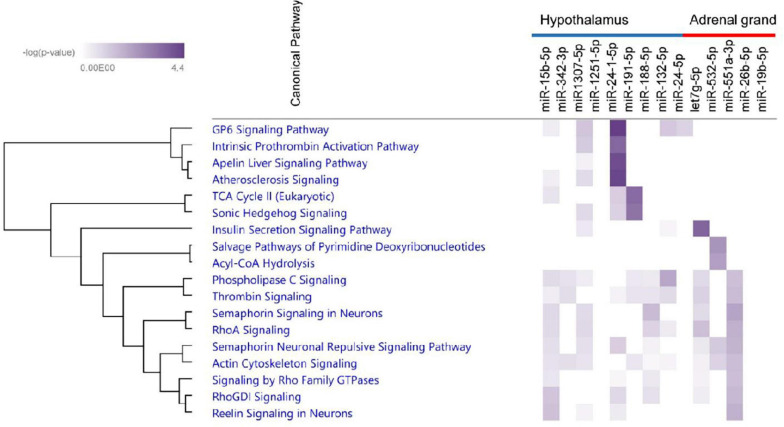
Heatmap showing enriched canonical pathways derived from analyses of miRNAs and corresponding mRNAs. The first nine miRNA including miR-15b-5p, miR-342-3p, miR1307-5p, miR-125-1-5p, miR-24-1-5p, miR-191-5p, miR-188-5p, miR-132-5p, and miR-24-5p belong to bio-signature miRNA of the hypothalamus. The other five miRNA belong to bio-signature miRNA of the adrenal grand. The intensity of color indicates significance from light to dark.

**FIGURE 12:**
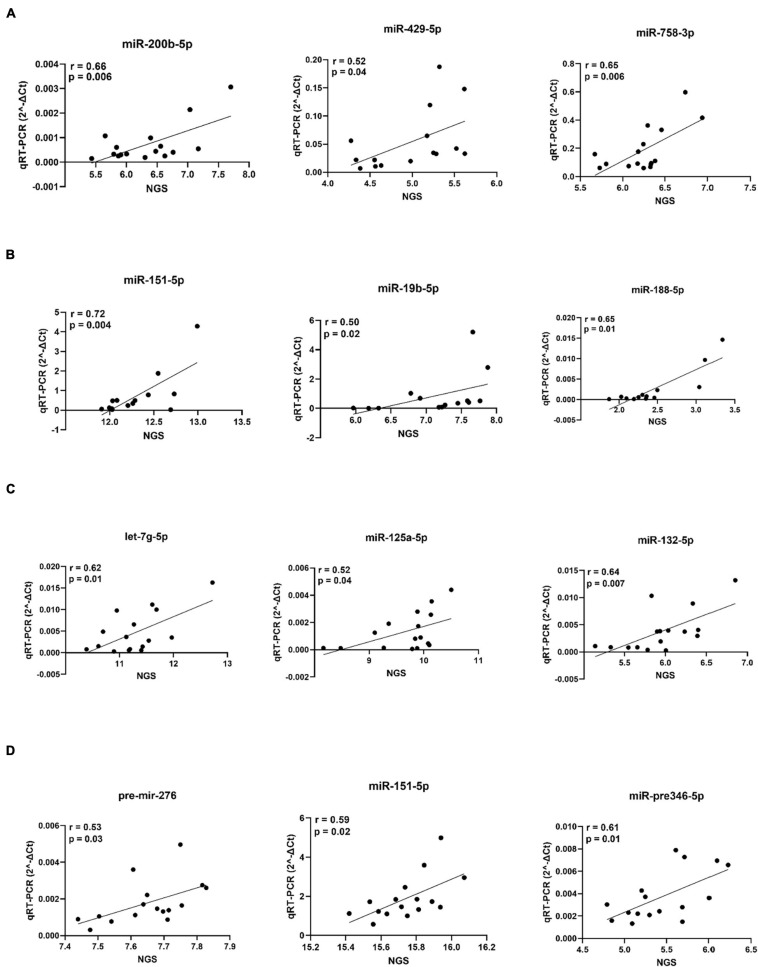
qPCR validation of selected miRNAs of the adrenal gland **(A)**, amygdala **(B)**, hippocampus **(C)** and hypothalamus **(D)**. For each miRNA, variance stabilized transformed NGS signals are plotted on the *x*-axis and qPCR (2^–ΔCt) data on the *y*-axis.

We further selected the miRNA bio-signature for target prediction. In this part, we used the identified miRNAs and all transcripts that negatively correlated with these miRNAs and were predicted to be putative targets. With this analysis, we can see the pathways that were regulated by the identified miRNAs. Sus scrofa genome (Sscrofa11.1) was used for the prediction of miRNA targets. Functional network analysis using IPA software was conducted in order to get biological insights into the interaction of the miRNAs and their predicted targets. After combining the correlation analysis and target prediction results, 9 miRNA (miR-15b-5p, miR-342-3p, miR1307-5p, miR-125-1-5p, miR-24-1-5p, miR-191-5p, miR-188-5p, miR-132-5p, and miR-24-5p) from hypothalamus target in total 1,290 transcripts (Supplementary Table 3). The number of targets of individual miRNAs ranged from 2 to 591 transcripts. The associated biological pathways of each miRNA target was shown in Figure 11. Interestingly, the target transcripts of miR-188-5p and miR-15b-5p enriched in Semaphorin Signaling in Neurons. Additionally, miR-15b-5p targets were enriched in Reelin Signaling in Neurons. Targets of miR-1307-5p and miR-24-1 were enriched in GP6 Signaling Pathway. Six miRNA (let7g-5p, miR-532-5p, miR-551a-3p, miR-26b-5p, miR-370-3p, and miR-19b-5p) of bio-signature sets from the adrenal gland target 82 transcripts (Supplementary Table 4). The number of miRNA targets ranged from 1 in case of miR-370-3p to 56 transcripts in case of let7g-3p. The biological pathways of each miRNA target was shown in Figure 11. The targets of let7g-3p were enriched in Insulin Secretion Signaling Pathway, while targets of miR-551a-3p were enriched in Semaphorin Signaling in Neurons. At a significance level of FDR < 0.05, no targets from bio-signature miRNA of amygdala and hippocampus were found. Finally, we selected 3 miRNAs in each tissue for qPCR validation as shown in Figure 12. Our NGS miRNA data and qPCR miRNA data of the identical samples showed good consistency with the coefficient of correlation ranging from *r* = 0.50 to *r* = 0.72 at significance level *p* < 0.05.

## Discussion

Technological advances have allowed the collection of data from different layers of transcriptomic complexity, resulting in multiple omics (multi-omics) data obtained from the same set of samples. In our previous studies, using microarray technology, we identified mRNA transcriptomic profiles of the hypothalamic-pituitary-adrenal (HPA) axis (hypothalamus and adrenal gland) and the limbic forebrain system (amygdala and hippocampus), that differed between HR and LR groups enriched the molecular signaling pathways, and candidate genes underlying coping behavior in pigs (Gley et al., 2019a,b). In addition, using NGS sequencing technology, we reported small non-coding RNA (sncRNA) expression in central parts of the physiological stress and anxiety response system (Haack et al., 2019). Particularly, we observed marked differences in the expression profiles of limbic system tissues compared to those associated to the HPA/stress axis, with a surprisingly high aggregation of 3′-tRNA halves (3′-tiRNA) in amygdala and hippocampus (Haack et al., 2019). Potential causes for tiRNA formation are stressors like tissue hypoxia, nutrient deprivation, oxidative dysbalances, and metabolic abberations, where levels of tiRNAs are correlated with the degree of tissue damage (Anderson and Ivanov, 2014). Human studies showed a cell type and phenotype specific composition and abundance of tRFs and tiRNAs, which raises the possibility of using them as biomarkers (Telonis et al., 2015).

By incorporating data from different layers of biological complexity, system biology approaches provide improved biological insights compared to traditional single data analyses. Single omics analyses omit the interactions occurring between different omic layers and as a consequence, prevent the reconstruction of accurate molecular biological networks. Therefore, integrating multi-transcript level data in a holistic approach may bridge the information gaps, provide deeper knowledge about the transcriptional interplay shaping behavioral traits and uncover molecular networks underlying complex phenotypes like coping behavior. In the present study, the integrative analysis of mRNA, miRNA and tRNA revealed molecular drivers explaining the variation between the HR and the LR coping behavior group. The deduced molecular panels conveyed novel insights into various RNA classes building behavior-linked regulatory networks. We demonstrated potential mRNA-sncRNA interactions occurring in the animals that represent the extremes for varying coping behavior phenotypes.

Two microarray probe sets of the *ATP1B2* transcript led the list of top contributors on component 1, block “Mrna” in the adrenal gland. This gene encodes the ATPase Na+/K+ transporting subunit beta 2. Network visualization demonstrates a negative correlation between *ATP1B2* and miR-26b-5p and let-7g-5p, prompting the assumption of regulatory miRNA-mRNA-interaction between these RNAs. Furthermore, the tRNA^*Arg*^ and tRNA^*GlyiF*^ correlated positively to miR-19b-5p, miR-26b-5p, and let-7g-5p, whereas the tRNA halve 5′-tiRNA^*Leu*^ was negatively correlated. microRNAs and tRFs share many functional features including biogenesis in a Dicer-dependent manner, RNA silencing as well as RISC complex formation with Argonaute proteins (Kumar et al., 2014). Even direct mapping of mirBase cataloged miRNAs to tRF sequences has been demonstrated (Venkatesh et al., 2016). In our present study we showed miRNA and mRNA correlation of tRNA^*Arg*^, tRNA^*GlyiF*^ and 5′-tiRNA^*Leu*^ suggesting the assumption that they play a particular role as regulators of gene expression and as signaling molecules in the different coping behavior haplotypes.

The mRNA of Mannose-P-dolichol utilization defect 1 (*MPDU1*) gene followed *ATP1B2* in the list of top contributors on component 1 in the adrenal gland, explaining the LR group. The adrenal gland network showed negative correlation of *MPDU1* with miR-26b-5p, miR-19b-5p, tRNA^*Arg*^, tRNA^*GlyiF*^, let-7g-5p suggesting a functional interplay of these coping behavior linked biomarker molecules. Emerging evidence of miR-19-5p as a stress biomarker in relation to stressful events such as piglet castration and tail docking (Lecchi et al., 2020) and even as a widespread pain and posttraumatic stress symptom biomarker is accumulating (Linnstaedt et al., 2020). Negative correlation of 5-tiRNA^*Lys*^ as well as positive correlation of 5′-tRF^*Ala*^ and 5′-tiRNA^*Leu*^ with *MPDU1* was observed. *MPDU1* is required for the synthesis of both lipid-linked oligosaccharides (LLOs) and glycosylphosphatidylinositos (GPI) which previously have been linked to traits like porcine disease resistance (Yang et al., 2005) and might also play a role in coping behavior. MiRNA-mRNA target prediction revealed that 56 mRNAs in the adrenal gland samples are predicted targets of let-7g-5p. Functional pathway analysis highly significantly enriched these mRNA targets in the Insulin Secretion Signaling Pathway (Figure 11). This finding is in line with previous studies which identified the let-7 family as a central regulator of mammalian glucose metabolism (Zhu et al., 2011; Katayama et al., 2015; Ponsuksili et al., 2017). Dysregulation of this pathway was associated with impaired glucose tolerance and insulin resistance (Zhu et al., 2011; Katayama et al., 2015; Ponsuksili et al., 2017). Although the exact function of insulin in the brain and its effect on behavior is not well-understood, insulin secretion is known to modulate reproduction, feeding and cognition (Gerozissis, 2003; Erion and Sehgal, 2013).

Potential mRNA targets of miR-551a-3p in the adrenal gland were associated with Acyl-CoA Hydrolysis Signaling by IPA. Recent studies suggest that acetyl-CoA represents a sentinel metabolite which acts as key indicator of the cellular metabolic state (Shi and Tu, 2015). Cellular growth states are marked by a high nucleocytosolic acetyl-CoA level promoting its use for lipid synthesis and histone acetylation whereas low acetyl-CoA amounts promote ATP and ketone body synthesis (Shi and Tu, 2015). Importantly, the lipid cholesterol is synthesized from acetyl-CoA and acts as starting material for adrenal steroidogenesis (Baba et al., 2018). It is tempting to assume that miR-551a-3p and its predicted mRNA targets affect coping behavior by influencing acyl-CoA signaling and ultimately adrenal steroidogenesis.

By using the approach of integrating multi-level transcript data in order to identify key molecular drivers, we confirmed the results of our previous study showing that *ATP1B2* and *MPDU1*, genes located in the same prominent coping behavior haplotype associated QTL region on SSC12 and differentially expressed between both groups, represent meaningful molecules for coping behavior in the adrenal gland (Gley et al., 2019b). In addition miRNA and tRNA as well as their cleavage products were identified as molecules linked to coping behavior in the adrenal gland.

Interestingly, component 1 of the amygdala was marked by enrichment of 5′-tiRNA and 5′-tRFs which were associated with the LR group and included 5′-tRF^*Lys*^, 5′-tiRNA^*Lys*^, 5′-tiRNA^*Cys*^ and 5′-tiRNA^*Gln*^. In contrast to that, the HR group was marked by enrichment of 3′-tRFs including 3′-tRF^*Asn*^, 3′-tRF^*Glu*^ and 3′-tRF^*Val*^. A previous cell culture study reported that 5′-tiRNA halves including 5′-tiRNA^*Cys*^ enhanced stress granule formation, which were shown to be comparatively more potent translation repressors than other 5′-tiRNA species (Ivanov et al., 2011). In addition, the accumulation of 5′-tiRNA halves resulted in translation repression, activation of cell stress pathways and increased neuronal apoptosis (Blanco et al., 2014). tRNA-derived small RNA fragments (tRFs) were shown to be capable of transcription repression induction (Goodarzi et al., 2015). Here we found the tRNA fragment 5′-tRF^*Lys*^ belonging to the LR group linked molecular markers that is located in the coping behavior associated QTL region on SSC12. In the amygdala the strongest contribution on component 2, block mRNA was exerted by *IGF1*. The associated outcome is the HR group, showing negative correlation. In the compiled network, *IGF1* is negatively correlated to miR-58-5p, and positively correlated to 3′-tRF^*Val*^ and 3′-tRF^*Glu*^. IGF-1 signaling is widely known to play a role in the regulation of systemic metabolic processes especially via the hypothalamus (Soto et al., 2019). Additionally, studies in mice showed IGF-1 receptors in hippocampus and amygdala (Soto et al., 2019). Inactivation of these IGF-1 receptors resulted in decreased levels of the GluA1 subunit of the glutamate AMPA receptor leading to increased anxiety-like behavior and impaired cognition (Soto et al., 2019). In the model organism Caenorhabditis elegans insulin/IGF-1 signaling activity was shown to regulate the expression of members of the miR-58 microRNA family, indicating that these miRNAs are part of the extended IGF-1 signaling network (Zhang et al., 2018). In contrast to IGF-1 mRNA, expression of *APOD* was positively correlated to the HR group. The corresponding protein, apolipoprotein D (APOD), is distributed throughout the central and peripheral nervous system, where it has been shown to increase during neuro-regenerative processes as well as neurodegenerative and neuropsychiatric disorders (Rajput et al., 2009). ApoD(−/−) mice exhibited decreased expression of the neurotransmitter and neuromodulator somatostatin (SST) in cortex and hippocampus and increased SST expression in striatum and amygdala (Rajput et al., 2009). In a study with humans, significantly elevated apoD levels have been observed in the amygdalae of subjects with schizophrenia (Thomas et al., 2003).

Pre-B-cell leukemia homeobox transcription factor 1 (*PBX1*) was associated with the HR outcome in the hippocampus *plot loadings ()* results, contribution on component 1, block mRNA. A novel transcriptional network under the control of *PBX1* has been shown to be required for midbrain dopaminergic specification and survival (Villaescusa et al., 2016). *PBX1* is responsible for the protection of dopaminergic neurons from oxidative stress and reduced levels of nuclear *PBX1* were associated with Parkinson’s disease in humans. miR-194a-5p contributed on component 1, block miRNA in the hippocampus *plot loadings ()* results and was linked to the LR outcome. Transfection of miR-194a-5p mimics into human cortical neurons increased neuronal death in stressed (oxygen-glucose deprivation) cells (Wang et al., 2018). Another mRNA associated with the HR outcome in the same loadings plot is *TOB1*. The mRNA of *TOB1* showed positive *plot network ()* positive correlation to 3′-tRF^*Phe*^ and negative correlation to tRNA^*Ser*^ as well as miR-194a-5p, suggesting common post-transcriptional regulation to some extent in the specific coping behavior groups. Moreover, *TOB1* was positively correlated to 3′-tiRNA^*Phe*^ and miR-125a-5p. In the brain, *TOB1*transcripts have been specifically detected in the hippocampus and their function has been associated with learning and memory (Jin et al., 2005). Transient increases in Tob1 protein expression could be detected after behavioral training of fear conditioning (Jin et al., 2005). Furthermore, Tob1 has been linked to TGFß family mediated signaling and regulation of transcription (Baranzini, 2014). Interestingly, another gene in the hippocampus network, *C18orf1*, has been identified as a novel regulator of TGFß signaling (Nakano et al., 2014). Further top miRNA which belonged to the marker molecule included miR-7-1-5p, miR-107-5p, and miR-125a-5p. These miRNAs were also reported to be involved in schizophrenia and Alzheimer’s disease (miR-7-5p) (Zhang et al., 2015; Puthiyedth et al., 2016), or as potential biomarkers for Acute Ischemic Stroke (miR-125a-5p) (Tiedt et al., 2017). In our *network ()* results *C18orf1* correlated negatively to miR-7-1-5p and tRNA^*Glu*^ and—like TOB1—positively to 3′-tRF^*Phe*^. The central role of 3′-tRF^*Phe*^ in the hippocampus deserves further investigation.

Interestingly, we discovered 3′-tiRNA enrichment in the hypothalamus of the HR group including 3′-tiRNA^*Gln*^, 3′-tiRNA^*Asn*^, 3′-tiRNA^*Val*^, 3′-tRF^*Pro*^, 3′-tiRNA^*Cys*^, and 3′-tiRNA^*Ala*^. In comparison, the amygdala showed enrichment of 5′-tiRNA related to the LR group. Such specific 3′-/5′-tiRNA profiles linked to coping behavior were not found in the adrenal gland. Indeed, our previous study revealed 3′-tiRNA enrichment in amygdala and hippocampus while expression in hypothalamus and adrenal gland was decreased (Haack et al., 2019). Similar expression levels for 5′-tiRNAs was observed across all tissues (Haack et al., 2019). These evidence suggest that tRNA-derived fragments/halves and their cleavage activity are not only tissue-specific but also a specific marker for coping behavior in the brain. Knowledge about the regulatory impact of individual tRNA fragments/halves is still limited. In the hypothalamus, miR-24-5p contributed to the HR group on component 1, block miRNA. This miRNA was negatively correlated to *CBL* in the *network ()* plot. By identifying negative miRNA-mRNA correlation pairs and carrying out target prediction analysis, we confirmed that *CBL* was a target transcript of miR-24-5p. MicroRNA profiling in mice hypothalami found evidence for miRNA-mediated neurohypophysial hormone regulation and highlighted an oxytocin-regulating function of miR-24 (Choi et al., 2013). Oxytocin is produced in the hypothalamic paraventricular and supraoptic nuclei and it modulates stress responses and social behavior including affiliative behavior and maternal aggression to defend their offspring (Bosch, 2013; Ebert and Brüne, 2018; Winter and Jurek, 2019).

Furthermore in the part of miRNA bio-signature and target prediction, target prediction for miR-188-p and miR-15b-5p followed by subsequent pathway analysis enriched the putative target transcripts in Semaphorin Signaling in Neurons. Due to the vast number and diversity of semaphorins, the list of physiological processes that are controlled by semaphorin signaling is continuously growing. In the nervous system, semaphorins regulate neuronal proliferation and migration, help in neural polarity determination, regulate the function and formation of synapses and shape dendrite morphology (Pasterkamp and Giger, 2009). Owing to their widespread physiological functions, semaphorins have been linked to several neurological diseases including schizophrenia (Fujii et al., 2011) and anxiety disorder (Pasterkamp and Giger, 2009). Hence, it appears very likely that semaphorin signaling plays an important role in stress response and coping behavior, too. In addition, miR-15b-5p targets mRNA transcripts which were enriched in the Reelin Signaling in Neurons pathway. In the brain, activation of reelin signaling promotes enhancement of long-term potentiation, cell proliferation, cell migration as well was dendritic spine morphogenesis (Fatemi, 2011). Multiple studies have implicated underactive reelin signalin in the etiology of several neuropsychiatric disorders, including schizophrenia, bipolar disorder, autism and major depressive disorder (Impagnatiello et al., 1998; Fatemi, 2005). Reelin overexpression in a transgenic mouse model showed an anxiety-reducing, anti-depressant effect, as well as a reduction in psychotic and autistic behaviors (Teixeira et al., 2011).

In order to validate NGS generated miRNA data, we selected 3 miRNAs in each tissue and subjected them to qPCR analysis. Correlation of NGS and qPCR miRNA data ranged between *r* = 0.50 and *r* = 0.72 (*p* < 0.05), emphasizing reliable NGS data quality.

Finally, all molecular biomarkers linked to the coping behavior haplotypes were mapped to the porcine genome. We found interesting molecular markers in this study, which are located in the coping behavior associated QTL region of our previous study (Gley et al., 2019b; Haack et al., 2019). These molecular panels encompassing *MPDU1*, *ATP1B2*, *SNORD65*, 5′-tRF^*Lys*^, and *METTL16*, were associated with the LR group, while miR-132-5p and 3′-tiRNAs were associated with HR coping behavior.

## Conclusion

The study reveals new insights into the regulatory networks of RNAs of different classes including mRNA, miRNA, and tRNA-derived fragments/halves in four tissues relevant to coping behavior. We confirmed the previously demonstrated role of *ATP1B2* and *MPDU1* in the adrenal gland and added complementary mRNAs related to coping behavior included *IGF1* and *APOD* in the amygdala, *CBL* and *PVRL1* in the hypothalamus as well as *PBX1*, *TOB1*, and *C18orf1* in the hippocampus. Further we pointed out potential regulatory roles of miR-19b-5p, miR-26b-5p, and let-7g-5p as well as tRNA^*Arg*^, tRNA^*GlyiF*^ and 5′-tiRNA^*Leu*^. Further interesting coping behavior-haplotype-related miRNAs encompassed miR-58-5p in the amygdala as well as miR-194a-5p, miR-125a-5p, miR-7-1-5p, and miR-107-5p in the hippocampus. Additionally, we revealed evidence for miRNA-mediated neurohypophysial hormone regulation by miR-24 in the hypothalamus. We found evidence of tRNA-derived fragments/halves as specific markers for coping behavior in the brain. Most prominently, the central role of 3′-tRF^*Phe*^ in the hippocampus deserves further attention. Hypothalami of the HR group were recognizably marked by 3′-tiRNA enrichment including 3′-tiRNA^*Gln*^, 3′-tiRNA^*Asn*^, 3′-tiRNA^*Val*^, 3′-tRF^*Pro*^, 3′-tiRNA^*Cys*^, and 3′-tiRNA^*Ile*^. Our study demonstrated that amygdala and hippocampus harbor a high aggregation of 3′-tRNA halves, while specific 3′-/5′-tiRNA profiles linked to coping behavior were absent in the adrenal gland. Detailed knowledge about the individual regulatory role of tRNA cleavage products still remains subject of future research. Small non-coding RNAs like miRNAs, tRNAs and their cleavage products play an intricate role as gene expression regulators and signaling molecules in the different coping behavior haplotypes. Our integrative analysis of mRNA and sncRNA data on different transcripts levels in the four tissues adrenal gland, amygdala, hippocampus and hypothalamus revealed new bio-signatures which explained the variation between the high reactive and low reactive coping styles as the extremes for varying early life coping behavior phenotypes.

## Data Availability Statement

The raw data was deposited in the NCBI Gene Expression Omnibus (www.ncbi.nlm.nih.gov/geo) (GEO: GSE109155) and ArrayExpress with the accession number (E-MTAB-7499).

## Ethics Statement

The animal study was reviewed and approved by animal care and tissue collection procedures followed the guidelines of the German Law of Animal Protection and the experimental protocol was approved by the Animal Care Committee of the Leibniz Institute for Farm Animal Biology as well as by the State Mecklenburg-Western Pomerania (Landesamt für Landwirtschaft, Lebensmittelsicherheit und Fischerei; LALLF M-V/TSD/7221.3-2.1-020/09).

## Author Contributions

SP and KW: conceptualization. NT, FrH, FiH, and KG: data curation. KG: formal analysis, visualization and writing—original draft. EM, KG, and NT: investigation. SP: methodology, resources and supervision. SP, KW, NT, EM, and UG: writing—review and editing. All authors have read and agreed to the published version of the manuscript.

## Conflict of Interest

The authors declare that the research was conducted in the absence of any commercial or financial relationships that could be construed as a potential conflict of interest.

## Publisher’s Note

All claims expressed in this article are solely those of the authors and do not necessarily represent those of their affiliated organizations, or those of the publisher, the editors and the reviewers. Any product that may be evaluated in this article, or claim that may be made by its manufacturer, is not guaranteed or endorsed by the publisher.

## Footnotes

1
www.ncbi.nlm.nih.gov/geo

2
https://rnacentral.org
